# Comparison of Antioxidant Properties of Fruit from Some Cultivated Varieties and Hybrids of *Rubus idaeus* and *Rubus occidentalis*

**DOI:** 10.3390/antiox14010086

**Published:** 2025-01-13

**Authors:** Natalia Adamczuk, Mirosława Krauze-Baranowska, Justyna Ośko, Małgorzata Grembecka, Piotr Migas

**Affiliations:** 1 Department of Pharmacognosy with Medicinal Plant Garden, Faculty of Pharmacy, Medical University of Gdańsk, 80-416 Gdańsk, Poland; natalia.adamczuk@gumed.edu.pl (N.A.); piotr.migas@gumed.edu.pl (P.M.); 2 Department of Bromatology, Faculty of Pharmacy, Medical University of Gdansk, 80-416 Gdańsk, Poland; justyna.osko@gumed.edu.pl (J.O.); malgorzata.grembecka@gumed.edu.pl (M.G.)

**Keywords:** *Rubus occidentalis*, *Rubus idaeus*, cultivars, hybrids, fruits, antioxidants, chemometric analysis

## Abstract

The aim of this study was to compare the antioxidant potential in the fruits of different hybrids of *Rubus idaeus* and *Rubus occidentalis* (four hybrids) against the fruit of known cultivars of both species (*R. idaeus*—three cultivars; *R. occidentalis*—five cultivars) and, using chemometric analysis, to select factors affecting the level of polyphenols and antioxidant properties. Antioxidant activity was determined using the ABTS, DPPH and FRAP tests. Chemometric analysis enabled the separation of *R. idaeus* and *R. occidentalis* cultivars and classified the hybrid *R. idaeus*/*R. occidentalis* R1314701 as belonging to the *R. occidentalis* species. Moreover, two hybrids, *Rubus occidentalis*/*Rubus idaeus* R1613411 and *R. idaeus*/*R. occidentalis* R1613409, can be classified as a purple raspberry. Crossbreeding species/cultivars of the *Rubus* genus may result in an increased content of anthocyanins, but on the other hand, it may lead to a reduction in free radical scavenging activity in the ABTS and DPPH. Spearman’s correlations confirm the correlations between the total polyphenol content and antioxidant activity in the DPPH, ABTS and FRAP, as well as the anthocyanin content and antioxidant activity in the ABTS and FRAP tests. Chemometric analysis can be an effective tool in determining the species affiliation of obtained hybrids and cultivars.

## 1. Introduction

The fruits of many raspberry species are known for their antioxidant properties, which have been evaluated in various in vitro [1,2,3,4,5,6,7,8,9,10,11,12] and in vivo [13,14] models. In addition, for the fruits of two species of *Rubus idaeus* L. (red raspberry) and *Rubus coreanus* L. (black raspberry), a species similar in chemical profile to *Rubus occidentalis* (black raspberry), antioxidant activity has been confirmed in clinical studies [15]. In recent years, various publications have compared the antioxidant activity of the fruits of some varieties of *R. idaeus* [1,2,3] and *R. occidentalis* [3,9,12,16] and evaluated *R. chamaemorus* L. [17,18,19] and *R. coreanus* L. [20,21]. The mentioned species belong to the genus *Rubus* and subgenus *Ideobatus*, in contrast to *Rubus fruticosus* L., which belongs to the subgenus *Rubus* [22]. The fruits of *Rubus occidentalis* were found to have the highest antioxidant activity among extracts obtained from fruits of *Rubus laciniatus* L., *Rubus ursinus* L., *Rubus ursinus x idaeus* and *Rubus idaeus* species [23].

Raspberry fruits are considered a rich source of polyphenolic compounds. For raspberry fruits, the highest levels of these compounds, about 500 mg/100 g fresh weight, were recorded for fruits of some *R. idaeus* and *R. occidentalis* varieties. These values are lower than those described for blackberry fruit (758 mg/100 g) and higher than those described for blueberry (305 mg/100 g) or cherry (314 mg/100 g) [24]. The complex of polyphenols in raspberry fruit consists not only of anthocyanins but also of ellagitannins, flavonoids, flavan-3-ols and phenolic acids. However, other compounds from the flavonoid group, namely flavan-3-ols (monomeric and dimeric forms) and also simple phenols—phenolic acids—are present in significantly lower concentrations. Black raspberries (*R. occidentalis*) were found to contain about 2–5 times more anthocyanins compared to red raspberries (*R. idaeus*) [25]. In both the anthocyanin complexes of red raspberry fruit and black raspberry fruit, cyanidin derivatives are present as the dominant ones alongside pelargonidin derivatives present in significantly lower concentrations. The compounds characteristic of the fruits of various raspberry species are mainly ellagitannins, which are absent in other berries. Kula et al. [26] showed significant differences in the content of ellagitannins in the fruit of some cultivated varieties of *R. occidentalis* and *R. idaeus*, especially sanguiin H-6 as the dominant compound in the complex, respectively: 1537.2 mg/100 g and 899.4–1743.6 mg/100 g.

In addition to polyphenols, antioxidants present in raspberry fruits include some elements that are cofactors of enzymes involved in the free radical scavenging reaction, including copper, iron, zinc and manganese. Their presence has been demonstrated in the fruit of some cultivated varieties of *R. occidentalis* and *R. idaeus* and their hybrids [27].

The level of antioxidant content in raspberry fruit can be influenced by a number of factors, including species, cultivar, fruit ripening stage, soil and climatic conditions. These factors help explain the differences observed in polyphenol concentrations in annually compared growing cycles [9]. Reactive oxygen species (ROS) are a major contributor to damage at the cellular level in diseases such as cancer, cardiovascular disease, diabetes, rheumatoid arthritis and neurodegenerative diseases [28].

Most ROSs are formed in the cell as a result of normal cellular metabolism. However, their levels can be increased as a result of the influence of various environmental factors, such as ultraviolet light, the presence of toxic chemicals and the inflammatory process. Capturing and scavenging ROSs is one of the main roles of antioxidants of natural origin [29].

The antioxidant properties of raspberry fruit have been confirmed in a number of in vitro tests. The antioxidant potential has been evaluated in terms of scavenging DPPH (2,2-diphenyl-1-picrylhydrazyl) [1,2,3,4,5,6,7,8,9,10,11,12,16,30,31] and ABTS (diammonium salt of 2,2′-azino-bis (3-ethylbenzothiazoline-6-sulfonic acid) radical [2,3,5,8,11,12,30,32], as well as blocking free radical reactions—in FRAP (ferric reducing antioxidant power) [8,9,11,12] and CUPRAC (cupric ion reducing antioxidant capacity) tests [9].

Using the DPPH assay to assess the level of free radical scavenging, Huang et al. [16] revealed stronger antioxidant properties of black raspberry fruit compared to fruit of some cultivars of red raspberry and other berries, which was related to the level of anthocyanin content. Similarly, Wada and Ou [23] evaluated the antioxidant activity of fruits from different species of the genus *Rubus* using the ORAC (Oxygen Radical Absorbance Capacity) method in relation to the content of anthocyanin compounds.

The anthocyanins that differentiate red raspberry from black raspberry are cyanidin 3-*O*-xylosyltrutinoside, found in *R. occidentalis* as the dominant anthocyanin compound, and cyanidin 3-*O*-glucosylrutinoside, an anthocyanin compound present only in red raspberry fruit. In addition, in the fruits of *R. occidentalis* and *R. idaeus,* there are such compounds like cyanidin 3-*O*-rutinoside, cyanidin 3-*O*-sophoroside, cyanidin 3-*O*-sambubioside and pelargonidin 3-*O*-rutinoside. Cyanidin has the strongest antioxidant properties compared to the other anthocyanosides [33,34]. Two different free radical scavenging pathways/mechanisms have been demonstrated for anthocyanins, while ellagitannins use only one. The pathway used by all hydrolysable tannins is characterised by oxidative coupling of adjacent galloyl groups [35]. Anthocyanins are able to couple free radicals by transferring an electron from their hydroxyl groups located in the B ring and/or from an oxonium ion on the C ring of anthocyanins [36].

Several works have analysed the phytochemical profile and its effect on antioxidant potential [1,2,3,11,12]. The antioxidant properties of raspberry fruits are determined not only by the level of anthocyanin content but also by structural differences and the degree of hydroxylation and glycosidation of anthocyanosides. The antioxidant activity of anthocyanins increases as the number of sugar residues in the molecule of the compound decreases and is strongest for the free aglycone, cyanidin, which has hydroxyl groups at the C-3, C-5, C-7, C-3′ and C-4′ positions [37].

According to Beekwilder [38], the share of anthocyanins in the total antioxidant activity of fruits is about 25%. Moreover, ellagitannins—including sanguiin H-6 and lambertianin C—most strongly determine the antioxidant activity of the polyphenol complex from *Rubus idaeus* fruits, and it constitutes 50% of the antioxidant activity of raspberry fruits with the mentioned share of anthocyanins and vitamin C in a range of approximately 15–20% [39,40]. It has been established that sanguiin H-6 is responsible for the antioxidant activity of raspberry fruits in the range of 30–45% [38,41], while lambertianin C is responsible for 14% [38].

The correlation between antioxidant activity and total polyphenol content was found to be higher than that between total anthocyanin content [23,42,43].

On the other hand, anthocyanins from raspberry fruit show the strongest inhibitory effect on liposome membrane protein oxidation reactions among phenolic compounds present in berries. The hydrolysis product of ellagitannins, ellagic acid, is a free radical scavenger and inhibits LDL (low-density protein) oxidation. It also has antioxidant activity against liposomal proteins [4].

Considering the importance of raspberry fruits as dietary components in the prevention of civilisation diseases, breeders are constantly looking for new varieties with a higher content of polyphenolic compounds determining their antioxidant properties. The aim of this study was to compare the antioxidant potential in the fruit of different hybrids of *R. idaeus* and *R. occidentalis* against the fruit of known cultivars of both species and, using chemometric analysis, to select factors affecting the level of polyphenolic compounds and antioxidant properties.

## 2. Materials and Methods

### 2.1. Plant Material

The plant material comprised fresh fruits from four cultivar varieties of *Rubus occidentalis* L. (*Rosaceae*), namely ‘MacBlack’, ‘Jewel’, ‘Niwot’ and ‘Heban’ and four of its hybrids R1613411, R1613409, R1613412, R1314701; three cultivars of *Rubus idaeus* L. (*Rosaceae*); and two varieties of ‘Delniwa’ and ‘Husaria’ and its one hybrid R1616002. These were collected in July 2020 from plants growing in Niwa Berry Breeding Company (Brzezna, Poland). Fruits of the ‘Bristol’ variety were obtained from two producers: “Czarna malina” Barbara Rusiecka-Górniak (Nałęczów, Poland) and BiGrim Grzegorz Maryniowski (Łaziska, Poland) in July 2020 (Table 1). The tested fruits were frozen at −20 °C, freeze-dried and powdered. The plant material was stored without access to moisture at room temperature.

### 2.2. Preparation of Samples

An amount of 0.5 g of lyophilised and powdered fruits (±0.0001 g) was extracted using an acidified methanol (methanol:formic acid 99.9:0.1 *v*/*v*) (3 × 15 mL) in a sonic bath (25 °C, 10 min). Each extraction was followed by a centrifugation of the sample (20 min, 5000 RPM). The extract was decanted into a volumetric flask and made up to a volume of 50 mL with the extraction mixture.

### 2.3. Methods

#### 2.3.1. Analysis of Polyphenols Content by Spectrophotometric Method

To 200 µL of an acidified methanolic extract from the tested fruits of raspberry, cultivars diluted 1:3 with deionised water and 200 µL of Folin–Ciocalteu reagent were added and allowed to stand for 6 min. Then, 2 mL of a 7% aqueous solution of anhydrous Na_2_CO_3_ was added. The absorbance of the sample was measured after 90 min at 750 nm. The standard solution was a sample containing 200 µL of deionised water. A solution of gallic acid in acidified methanol containing 0.1% HCl at concentrations of 1, 2, 4, 5, 10 and 20 mg/50 mL was used to determine the standard curve on the day of analysis. The content of total phenolic compounds was expressed in [%] per gallic acid [44].

#### 2.3.2. Analysis of Anthocyanins Content by Spectrophotometric Method

The analysis was carried out according to the monograph *Myrtilli fructus recens* [45]. The absorbance of the test solution was measured at λ-528 nm using 0.1% methanol as a reference. The percentage of anthocyanins was determined per cyanidin chloride 3-*O*-glucoside by substituting the measurement result into the formula

c = (A × 500)/(718 × m)

where A—absorption of the sample at λ-528 nm, m—mass of raw material in grams and 718—specific absorption for cyanidin 3-*O*-glucoside chloride at λ-528 nm.

#### 2.3.3. Analysis via the Spectrophotometric Method of Antioxidant Activity

Analysis was carried out using 2,2-diphenyl-1-picrylhydrazyl (DPPH) using the diammonium salt of 2,2′-azobis (3-ethylbenzothiazoline-6-sulfonate) (ABTS) and determining the reducing ability of iron (III) ions (FRAP) [46,47,48]. The determination of antioxidant activity was carried out using methanol fruit extracts from analysed cultivars and hybrids and dilutions with methanol 1:50.

##### Stable 2,2-Diphenyl-1-picrylhydrazyl (DPPH•) Radical Test

The assessment of antioxidant activity was performed using a 0.04 mM methanol solution of the stable 2,2-diphenyl-1-picrylhydrazyl radical (DPPH•) against a standard curve prepared from six dilutions of 6-hydroxy-2,5,7,8-tetramethylchroman-2 acid -carboxylic acid (Trolox) in methanol with concentrations 0.02; 0.04; 0.05; 0.06; 0.08; and 0.1 mM. Before starting the measurements, the freshly prepared methanolic DPPH solution was incubated for 60 min at 4 °C. Then, 2.5 mL of DPPH solution and 350 μL of the extract from analysed fruits were combined in quartz cuvettes, the prepared mixtures were incubated for 30 min in a dark place at room temperature, and then spectrophotometric measurements were made at wavelength λ-517 nm.

##### Determination of the Ability to Reduce Iron (III) Ions—FRAP Test

The determination of the reducing capacity of iron (III) ions was carried out using the FRAP reagent, which consists of mixed in a ratio of 10:1:1 (*v*/*v*) and brought to a temperature of 37 °C:

- 300 mM acetate buffer solution, pH 3.6.
- 10 mM solution of 2,4,6-tris(2-pyridyl)-1,3,5-triazine (TPTZ) in 40 mM hydrochloric acid (HCl).
- 20 mM FeCl_3_ × 6H_2_O solution in water against a standard curve prepared from six dilutions of trolox in water at the following concentrations: 0.02; 0.03; 0.06; 0.12; 0.36; 0.48 mM.

Before starting the measurements, the FRAP reagent was heated in a water bath to 37 °C. Then, 3 mL of FRAP reagent and 150 μL of the extract from analysed fruits were combined in quartz cuvettes, the prepared mixtures were incubated for 30 min in a dark place at room temperature, and then a spectrophotometric measurement was performed at a wavelength λ-593 nm.

##### Determination of Antioxidant Activity Using 2,2′-Azobis(3-ethylbenzothiazoline-6-sulfonate) Diammonium Salt—ABTS Test

In order to determine the antioxidant activity, the ABTS •+ cation radical was generated by incubating the mixture in a dark place at 4 °C for 15 h:

- 2 mL of 7 mM solution of 2,2′-azobis(3-ethylbenzothiazoline-6-sulfonate) diammonium salt (ABTS).
- 0.35 mL of 140 mM potassium persulphate.

After the incubation time, the mixture was diluted with redistilled water in the ratio of 1:90 (*v*/*v*) while maintaining a constant absorbance of 0.7 ± 0.02. Measurements were carried out against a standard curve prepared from six dilutions of trolox in water at the following concentrations: 0.02; 0.03; 0.05; 0.08; 0.1; 0.12 mM.

An amount of 2 mL of ABTS reagent and 200 μL of the analysed sample were combined in quartz cuvettes. Then, the absorbance was measured twice at a wavelength of 734 nm 6 min after the start of the reaction.

#### 2.3.4. Analysis of Anthocyanin Content Using Chromatographic Methods—HPLC/HPTLC

In the studied plant material, anthocyanin contents were determined using HPLC/HPTLC methods, and the obtained results are included in the publication [25]. The content of anthocyanins in the analysed fruits was calculated as cyanidin equivalents and reported as the sum of the peak areas of these compounds. Both methods were validated in terms of a calibration curve, correlation coefficient, limit of detection, limit of quantitation and intra- and inter-day repeatability.

### 2.4. Statistical Analysis

Statistical analysis was based on data obtained from the following determinations: anthocyanin content by HPLC, HPTLC [25] and spectrophotometric methods separately, total polyphenols content (TPC—spectrophotometric), DPPH, ABTS, FRAP methods and content of individual substances (HPLC) such as cyanidin 3-*O*-rutinoside, cyanidin 3-*O*-xylosyl rutinoside, cyanidin 3-*O*-glucoside, pelargonidin 3-*O*-rutinoside and cyanidin 3-*O*-sambubioside. The distribution of data was non-parametric; thus, Kruskal–Wallis test and Spearman’s rank correlation were used. The analyses were performed using Statistica 13.3. The data were properly prepared by standardising them (columns—substance content, rows—raspberry samples). The use of factor analysis (FA) and cluster analysis (CA, Ward’s method; Euclidean distance) made it possible to differentiate raspberry samples according to their species origin in terms of their content of bioactive substances.

## 3. Results

### 3.1. Determination of Active Compounds in Fruits of the Genus Rubus

#### 3.1.1. Determination of Total Anthocyanin Content

Among the tested fruits of different black and red raspberry cultivars, the highest total anthocyanin content was found in fruits of *R. occidentalis* cultivars, namely ‘MacBlack’—2.89%, ‘Jewel’—2.58 and ‘Niwot’—2.36%. In the fruits of other black raspberry cultivars, the total anthocyanin content was lower, namely in the fruit of ‘Bristol’ in the range of 1.37–2.17%, while in the fruit of ‘Heban’ (1.67%). Fruits of all the hybrids studied were characterised by relatively high anthocyanin content, almost two to three times higher compared to fruits of red raspberry varieties. The highest anthocyanin content was detected in the fruits of the hybrid *R. occidentalis*/*R. idaeus* R1613412 at 3.66%. In the fruits of red raspberry, the anthocyanin content was 3–6 times lower compared to all tested varieties and hybrids (0.57–0.6%).

#### 3.1.2. Determination of Total Polyphenol Content

The total content of polyphenolic compounds was determined by the spectrophotometric method. The highest total content of polyphenols was revealed for the fruit of *R. occidentalis* cultivar ‘Bristol’ regardless of origin [’Bristol’ A (4.9%) ‘Bristol’ B (4.63%)]. The fruits of the other black raspberry cultivars had slightly lower polyphenol content [‘Heban’ (3.84%), ‘Jewel’ (3.8%), ‘MacBlack’ (3.72%) and ‘Niwot’ (3.7%)]. The highest content of polyphenolic compounds among hybrids was distinguished by the hybrid *R. occidentalis*/*R. idaeus* R1613412 (4.16%); against the others, the content of polyphenolic compounds was at a similar level (2.98–3.16%). Fruits of *R. idaeus* species contained two to three times lower content of polyphenolic compounds ‘Delniwa’ (1.76%), ‘Hussaria’ (1.56%) and R1616002 (1.15%).

Taking into account the differences between the determined total polyphenol content and the anthocyanin content, the levels of other polyphenolic compounds present in raspberry fruits—namely ellagitannins, flavonoids and flavan-3-ol (including proanthocyanidins)—were calculated. The highest content of these compounds was characterised by black raspberry fruits, including the cultivar ‘Bristol’ A (3.53%) and ‘Bristol’ B (3.26%). A relatively lower content was characterised by fruits of *R. occidentalis* cultivar ‘Heban’ (2.17%), followed by hybrids *R. occidentalis*/*R. idaeus* R1613411 (1.81%) and *R. idaeus*/*R. occidentalis* R1314701 (1.48%). Other fruits of hybrids were characterised by lower contents: *R. idaeus*/*R. occidentalis* R1613409 (0.97%), *R. occidentalis*/*R. idaeus* R1613412. The remaining black raspberry fruit varieties—’Niwot’ (1.34%), ‘Jewel‘ (1.24%) and ‘MacBlack’ (0.83%) varied in the content of the above-mentioned compounds. Fruit of *R. idaeus* species, depending on the variety, contained, respectively: ‘Delniwa’ (1.16%), ‘Husaria’ (0.99%) and R1616002 (0.56%).

### 3.2. Antioxidant Activity

The antioxidant activity of the tested raspberry fruits was evaluated using DPPH, ABTS and FRAP tests. The DPPH and ABTS tests are based on the ability to scavenge free radicals, while the FRAP test is based on the reduction of iron ions. The above tests followed the HAT (Hydrogen Atom Transfer) mechanism.

Among the tested fruits, the hybrid of *R. idaeus*/*R. occidentalis* R1314701 exhibited high antioxidant activity in all three assays, including the highest content in the ABTS test (2.31 mmol TE/g DW) In the DPPH test, the fruits of the *R. idaeus*/*R. occidentalis* hybrid R1314701 demonstrated activity comparable to the antioxidant activity of R. occidentalis ‘Bristol’ B, and similarly in the FRAP test, the activity of this hybrid was close to the antioxidant activity of the fruits of the studied R. occidentalis cultivars [3.01–4.29 mmol TE/g DW] (Table 2).

#### 3.2.1. DPPH

Among all tested fruits cultivars and hybrids *R. idaeus* and *R. occidentalis*, the highest antioxidant activity determined by using DPPH assay was in all black raspberry fruits (no statistically significant differences TAB), namely in cultivars ‘Bristol’ A (2.14 mmol TE/g DW), ‘Bristol’ B (1.95 mmol TE/g DW), ‘Jewel’ (1.82 mmol TE/g DW), ‘MacBlack’ (1.63 mmol TE/g DW), ‘Niwot’ (1.59 mmol TE/g DW), ‘Heban’ (1.53% mmol TE/g DW)—no significant statistical differences at the *p* = level. Fruits of red raspberry cultivars were characterised by two times lower antioxidant potential in the free radical scavenging test with DPPH reagent: ‘Hussaria’ (1.10 mmol TE/g DW), R1616002 (1.02 mmol TE/g DW), ‘Delniwa’ (0.97 mmol TE/g DW) (statistically significant difference). Among the tested hybrids, the hybrid *R. occidentalis*/*R. idaeus* R1613412 (1.03 mmol TE/g DW) and the hybrid *R. idaeus*/*R. occidentalis* R1613409 (1.16 mmol TE/g DW) had twice lower antioxidant potential of black raspberry fruit. The exception was the hybrids *R. occidentalis*/*R. idaeus* R1613411 (1.60 mmol TE/g DW) and *R. idaeus*/*R. occidentalis* R1314701 (1.95 mmol TE/g DW) that showed activity at the same level in comparison to black raspberry cultivars.

#### 3.2.2. ABTS

The free radical scavenging potential was also tested using the ABTS reagent. Among the two species tested, *R. occidentalis*, cultivars ‘Niwot’ (2.12 mmol TE/g DW), ‘Jewel’ (1.70 mmol TE/g DW), ‘MacBlack’ (1.60 mmol TE/g DW) had the highest activity. In the black fruit of *R. occidentalis* ‘Bristol’, there were no differences in antioxidant activity regardless of origin: ‘Bristol’ A and Bristol ‘B’ (1.24 mmol TE/g DW). The lowest antioxidant activity among black raspberries in the ABTS test was characterised by the cultivar ‘Heban’ (0.97 mmol TE/g DW). Fruits of red raspberry varieties were characterised by three and five times lower antioxidant activity [R1616002 (0.55), ‘Husaria’ (0.54), ‘Delniwa’ (0.27) mmol TE/g DW] in relation to black raspberry varieties. Fruits of all hybrids tested were characterised by relatively lower antioxidant activity than those of red raspberry cultivars, the exception being the hybrid *R. idaeus*/*R. occidentalis* R1314701 (2.31 mmol TE/g DW) with the highest antioxidant potential among all cultivars and hybrids tested.

#### 3.2.3. FRAP

The FRAP reagent test allowed the determination of antioxidant activity involving the reduction of iron ions. The highest antioxidant activity was observed for fruits of *R. occidentalis* cultivars, namely: ‘Bristol’ B (4.09 mmol TE/g DW), ‘Bristol’ A (3.87 mmol TE/g DW), ‘MacBlack’ (3.56 mmol TE/g DW) and ‘Jewel’ (3.21 mmol TE/g DW). The other cultivated varieties, ‘Niwot’ (2.73 mmol TE/g DW) and ‘Heban’ (2.47 mmol TE/g DW), were distinguished by the lowest activity among the tested black raspberry varieties. Among the tested hybrids, the hybrid *R. occidentalis*/*R. idaeus* R1613412 (3.79 mmol TE/g DW) and the hybrid *R. idaeus*/*R. occidentalis* R1314701 (3.52 mmol TE/g DW) had antioxidant potential similar to black raspberries. Fruits of *R. idaeus* species, as in other tests, were characterised by much lower antioxidant activity: ‘Husaria’ (1.49 mmol TE/g DW), ‘Delniwa’ (1.05 mmol TE/g DW) and R1616002 (1.03 mmol TE/g DW).

### 3.3. Correlation

Spearman’s rank correlations were performed at three levels of significance (*p* < 0.05; *p* < 0.01; *p* < 0.001). During the analysis, positive correlations (*p* < 0.001) were found to exist in almost the entire database analysed. Pairs in which no correlations occurred at the *p* < 0.001 were DPPH-anthocyanins HPLC, HPTLC, TPC (spectrophotometric), cyanidin 3-*O*-rutinoside, cyanidin 3-*O*-xylosyl rutinoside, cyanidin 3-*O*-glucoside, pelargonidin 3-*O*-rutinoside and cyanidin 3-*O*-sambubioside; cyanidin 3-*O*-sambubioside-TPC (spectrophotometric) and ABTS; FRAP-pelargonidin 3-*O*-rutinoside.

### 3.4. Kruskal–Wallis Test

The Kruskal–Wallis test was designed to show statistically significant differences in the analysed database in terms of the species origin of the raspberry samples. The following relationships were obtained: anthocyanins content by HPLC (H = 19.722; *p* = 0.0002), HPTLC (H = 19.450; *p* = 0.0002) and spectrophotometric method (H = 20.020; *p* = 0.0002), TPC by spectrophotometric method (H = 21.269; *p* = 0.0001), DPPH (H = 23.015; *p* = 0.0000), ABTS (H = 26.153; *p* = 0.0001), FRAP (H = 19.053; *p* = 0.0003) and individual substances content such as: cyanidin 3-*O*-rutinoside (H = 19.550; *p* = 0.0002), cyanidin 3-*O*-xylosyl rutinoside (H = 20.023; *p* = 0.0002), cyanidin 3-*O*-glucoside (H = 20.772; *p* = 0.0001), pelargonidin 3-*O*-rutinoside (H = 22.519; *p* = 0.0001) and cyanidin 3-*O*-sambubioside (H = 22.209; *p* = 0.0001).

### 3.5. Post Hoc Dunn’s Test

A Dunn’s post hoc test at three levels of significance (*p* < 0.05; *p* < 0.01 and *p* < 0.001) was performed to show the existence of statistical differences in the studied database. The results of the Dunn’s test analysis are presented in Table 3. Statistically significant differences were found for pelargonidin 3-*O*-rutinoside and cyanidin 3-*O*-sambubioside between the species *R. idaeus* and *R. occidentalis*/*R. idaeus*. The next relationships were for anthocyanins (HPLC), anthocyanins (HPTLC), anthocyanins (spectrophotometric), TPC (spectrophotometric), DPPH, ABTS, FRAP, cyanidin 3-*O*-rutinoside, cyanidin 3-*O*-xylosyl rutinoside, cyanidin 3-*O*-glucoside and pelargonidin 3-*O*-rutinoside between *R. occidentalis* and *R. occidentalis*/*R. idaeus* (*p* < 0.001).

### 3.6. Factor Analysis

**Figure 1:**
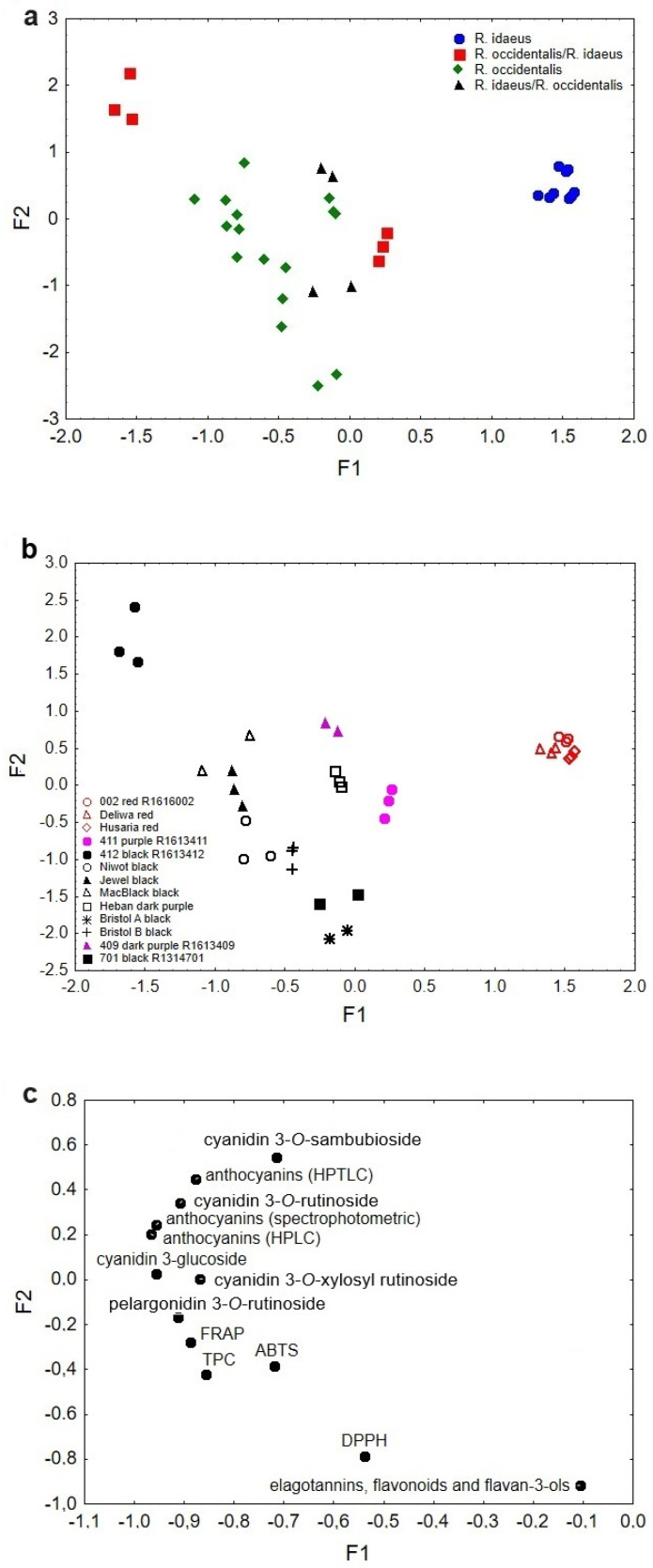
(**a**) Scatterplot of object samples of two factors in view of raspberry species. (**b**) Scatterplot of object samples of two factors in view of raspberry colours. (**c**) Scatterplot of loadings for elements in all the analysed samples.

Factor analysis was performed on all analysed raspberry samples. The results are presented in Figure 1a–c. All results are mentioned in Section 1. Statistical analysis was taken into account. The value of factor one (F1) explains 73.2% of the variance, and factor two (F2) explains 15.4%. The cumulative value of the explained variance of both factors is 88.6%. The eigenvalue for F1 was 8.79 and for F2 was 1.85.

As can be observed in Figure 1a, Factor 1 is responsible for the differentiation of the samples according to botanical species. Samples of *R. idaeus* raspberries were described with high values of F1, while those of *R. occidentalis*/*R. idaeus*—low ones. The mean F1 values describe samples of *R. occidentalis* and *R. idaeus*/*R. occidentalis*. Moreover, it is possible to observe a division of the samples of the hybrid species *R. occidentalis*/*R. idaeus* into two groups. The first one was described by high F1 values, indicating a higher proportion of *R. idaeus* species in this hybrid. The antioxidant potential determined by the DPPH method (using 2,2-diphenyl-1-picrylhydrazyl radical) is responsible for this differentiation. This discrimination between samples might be explained by the specificity of the method’s determination, in which the number of hydrophobic compounds is important. Other methods are characterised by the determination of compounds with only hydrophilic (FRAP and TPC) or both hydrophilic and lipophilic properties (ABTS) [49]. Low values of the F1 indicate a higher proportion of *R. occidentalis* species in the remaining raspberry hybrid samples of *R. occidentalis*/*R. idaeus* and *R. idaeus*/*R. occidentalis*, for which anthocyanins (HPLC, HPTLC and spectrophotometry), FRAP, TPC, cyanidin 3-*O*-rutinoside, cyanidin 3-*O*-xylosyl rutinoside, cyanidin 3-*O*-glucoside, pelargonidin 3-*O*-rutinoside and cyanidin 3-*O*-sambubioside are responsible for the differentiation (Figure 1c).

Factor 2 is probably responsible for the separation of raspberry samples in terms of increasing the amount of anthocyanins, the content of which mainly depends on the pH of the soil in which the plants are grown as well as the ambient temperature [50]. It is estimated that the darker the raspberry, the higher the anthocyanin content [51,52]. Therefore, F2 can differentiate raspberry samples according to their colour. Raspberries characterised by a lower anthocyanin content, with red or purple colour, were described by high F2 values (Figure 1b). Anthocyanins (HPLC, HPTLC, spectrophotometry) and compounds such as cyanidin 3-*O*-rutinoside, cyanidin 3-*O*-glucoside, cyanidin 3-*O*-xylosyl rutinoside and cyanidin 3-*O*-sambubioside are mainly responsible for this separation (Figure 1c). The low F2 values correspond to the dark-black raspberry samples. This group included raspberry samples from the species *R. occidentalis* and some from *R. idaeus*/*R. occidentalis*. TPC, FRAP, ABTS, DPPH and pelargonidin 3-*O*-rutinoside were responsible for this separation.

The factor analysis applied allowed samples to be differentiated mainly based on their botanical origin, i.e., species, and their content of colour-enhancing anthocyanin substances. The differentiation of the samples analysed is mainly related to their content of organic substances responsible for the antioxidant potential of raspberries.

### 3.7. Cluster Analysis

**Figure 2:**
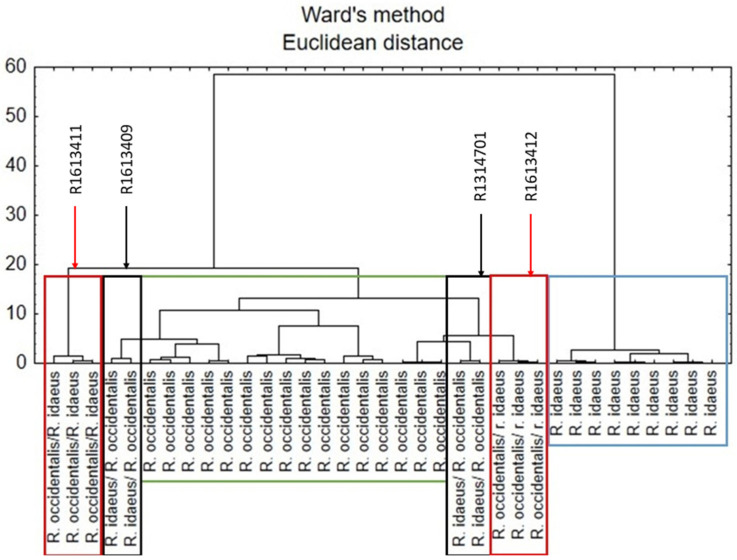
Hierarchical dendrogram for the raspberry species samples.

Cluster analysis was applied using Ward’s method and Euclidean distance. The dendrogram presented in Figure 2 shows the distribution of raspberry samples in view of their botanical provenance. There can be observed two main clusters. One of them contains samples only of *R. idaeus* species. However, the second one is divided into four subclusters, each of which contains one species/hybrid of raspberry.

## 4. Discussion

The antioxidant activity of fruits, determined using the DPPH, FRAP and ABTS assays, varied depending on the species/variety or hybrid tested and the method of assessing antioxidant properties (Table 2).

Most studies on the antioxidant activity of raspberries focus on the fruits of *Rubus idaeus* [1,2,6,8,9,10,11,12,16,32] and the evaluation of free radical scavenging properties using the DPPH assay [1,2,3,4,5,6,7,8,9,10,11,12]. These studies primarily involved the assessment of the antioxidant potential of juices from fresh fruits of various cultivars [1,2] and extracts obtained, among others, from frozen fruits [5,6,7,8,10,11,23,30,31,40]. In a few studies, the antioxidant properties of fruits after lyophilisation were analysed [3,4,9,11,12]. Fruits of various black raspberry cultivars, in comparative studies with red raspberry fruits using different assays, were generally characterised by stronger antioxidant activity—both in terms of scavenging free radicals and inhibiting their formation [9,10,11]. An exception was observed in the studies by Basu and Maier, where red raspberry fruits exhibited higher activity in the ABTS and FRAP assays compared to black raspberries. This suggests that the plant material studied (unknown cultivar) may contain compounds other than polyphenols responsible for this effect [12].

Studies on the antioxidant activity of black raspberries most commonly include the following cultivars: ‘Bristol’ [9,10,11,30], ‘Jewel’ [9,10,30,53], ‘MacBlack’ [30] or ‘Litacz’ [9]. In contrast, the antioxidant activity of fruits from red raspberry cultivars is more extensively studied. Literature data describing the antioxidant properties of other raspberry species examined are limited.

Viškelis et al. [10] assessed the antioxidant potential in the DPPH assay for fruits of various species and cultivars of raspberry, aronia and elderberry, revealing a strong correlation between antioxidant effect and total polyphenol content. The exception was the black raspberry variety ‘Bristol’, which accumulated relatively lower amounts of anthocyanins and polyphenols than the chokeberry and black elderberry fruits but showed only slightly lower free radical scavenging activity (approx. 9% lower and expressed as the mean RSA value) compared to the analysed *Aronia melanocarpa* fruits.

The antioxidant activity of raw extracts from six popular berry fruits was compared using the DPPH assay as an indicator of antioxidant activity. Black raspberry fruits exhibited the strongest antioxidant activity (2865.9 µg/mL) [53]. Wada et al. assessed the antioxidant activity using the ORAC method, for fruits of different *Rubus* species in relation to their anthocyanin content. It was found that extracts from *Rubus occidentalis* fruits, which have the highest polyphenol content, including anthocyanins, exhibited the highest antioxidant activity among the extracts from *R. idaeus*, *R. ursinus*, *R. laciniatus* and *R. idaeus* × *R. ursinus* species [23].

Similarly, in our studies using the DPPH assay, fruits from all the black raspberry cultivars tested exhibited higher antioxidant potential compared to the red raspberry cultivars (Table 2).

Some of the antioxidant activity results are expressed as % RSA—indicating the ability to eliminate free radicals [10], IC_50_—the concentration that inhibits 50% of free radicals [2,3,6,12,16] or EC_50_—the dose that inhibits 50% of free radicals [3,7]. Therefore, it is not possible to precisely compare the antioxidant potential between fruits of the same raspberry cultivars and species. Additionally, most of the results are converted to fresh fruit weight [1,5,8]. As a result, comparing the antioxidant potential of raspberry fruits from different cultivars and hybrids with published data is challenging. On the other hand, the literature data allow for the formulation of general conclusions about the mechanisms of antioxidant activity in raspberry fruits.

In three publications [8,11,12], similar to our study, the antioxidant activity of *R. idaeus* fruits was assessed using three assays, DPPH, ABTS and FRAP, with the highest antioxidant activity of red raspberries in each of these publications being observed in the FRAP assay. However, in the ABTS and DPPH assays, the measured antioxidant activity was highly varied depending on the origin of the fruits [2,3,8,10,11,12,30] and their drying method [11]. In our study, for the fruits of various cultivars and hybrids of two species, *R. idaeus* and *R. occidentalis*, the highest antioxidant activity was also observed using the FRAP assay (Table 2).

The antioxidant activity of black raspberry fruits was higher than that of red raspberry fruits in all the assays performed—DPPH, ABTS and FRAP. The results obtained were consistent with the literature data [9,11,12]. The fruits of black raspberry cultivars showed two to three times higher activity in reducing iron ions in the FRAP assay compared to free radical scavenging activity in the ABTS and DPPH assays (Table 2).

In summary, the results of the antioxidant tests showed that among the fruits studied, *R. occidentalis* ‘Bristol’ and the hybrid *R. occidentalis*/*R. idaeus* R1613412 exhibited stronger antioxidant activity in the FRAP assay—3.87–4.09 and 3.79 [mmol TE/g DW], respectively. At the same time, compared to the other fruits, *R. occidentalis* ‘Bristol’ showed high activity in the DPPH assay—1.95–2.14 [mmol TE/g DW], regardless of the place of origin.

Mitić et al. [54] demonstrated a correlation between anthocyanin content and the antioxidant activity of *R. idaeus* fruits. Kostecka-Gugała et al. found that the fruits of black raspberry (‘Bristol’, ‘Litacz’) with the highest total phenolic content (TPC) and total anthocyanin content exhibited the greatest antioxidant capacity. Some authors indicate that the determined antioxidant activity is influenced by extraction conditions, e.g., the type of solvent used for the extraction of raspberry fruits [9]. Kim et al. [20] revealed that a 50% ethanol extract from black raspberry fruits, with the highest total polyphenol and flavonoid content, exhibited the highest antioxidant activity in the DPPH and FRAP assays, while a 25% ethanol extract showed the highest antioxidant activity in the ABTS assay. In our research, we analysed methanol extracts from the tested fruits in all assays.

Red raspberry fruits, compared to other berries, are characterised by the highest share (70–80% of all phenols) in the chemical composition of compounds from the ellagitannin group, which in the in vitro model of LDL inhibition and methyl linoleate oxidation show the strongest antioxidant properties [18].

The contribution of other phenols, such as flavonols and phenolic acids, to the antioxidant activity of berry fruits, is significantly lower compared to the activity of anthocyanins. The anthocyanin profile of red raspberry fruits, consisting of cyanidin derivatives, exhibits the strongest free radical scavenging activity in the DPPH assay compared to anthocyanin profiles in other berry fruits [55].

**Figure 3:**
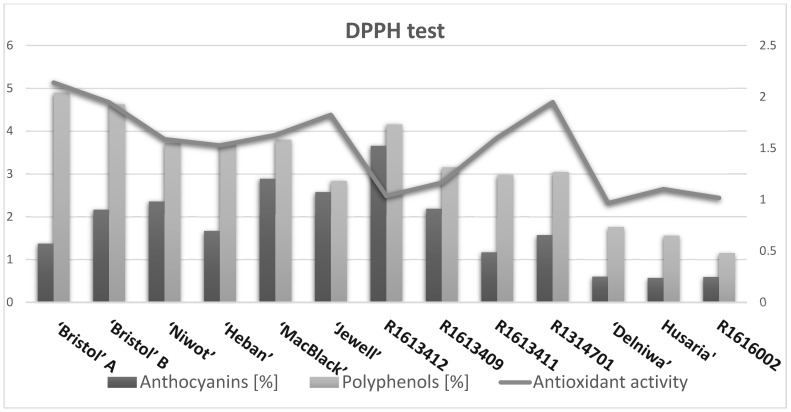

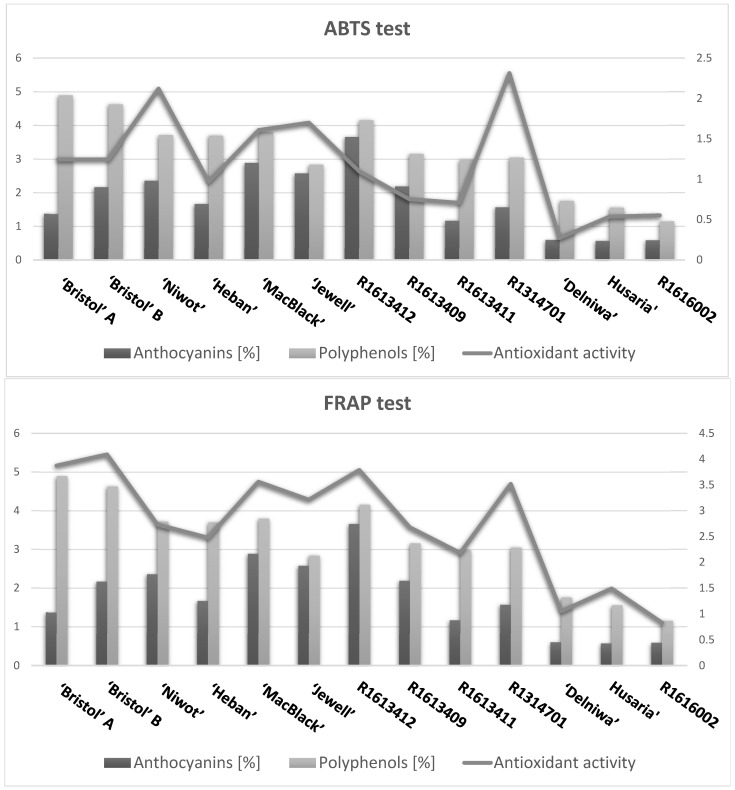
The determined antioxidant potential in the DPPH, ABTS and FRAP tests of the studied fruits of black and red raspberry cultivars and hybrids in relation to the determined content of polyphenolic and anthocyanin compounds.

Comparing the total polyphenol content in the studied raspberry fruits with the antioxidant properties determined in the DPPH and FRAP assays (Table 2 and Figure 3), it can be concluded that higher content of these compounds in the fruits increases antioxidant activity related to free radical scavenging and inhibiting their formation. An exception to this tendency is the hybrids *R. occidentalis*/*R. idaeus* R1613412 and *R. idaeus*/*R. occidentalis* R1314701. The first one is characterised by a high content of anthocyanins (ca. 90% in the complex of all compounds) and a low content of other polyphenolic compounds, such as ellagitannins, flavonoids and flavan-3-ol derivatives (ca. 10% in the complex of all compounds). At the same time, compared to the other fruits, it shows very low free radical scavenging activity in the DPPH and ABTS assays but one of the highest activities in the FRAP assay—inhibiting the free radical chain reaction. Conversely, the second hybrid is characterised by a relatively low total anthocyanin content (1.57%) and polyphenol content (3.05%), yet it exhibits the strongest activity in the ABTS assay and one of the strongest in the DPPH assay. The observed lack of correlation between the polyphenolic content in the fruits of the *R. idaeus*/*R. occidentalis* R1314701 hybrid and its high antioxidant potential in both assays suggests the involvement of other compounds besides polyphenols, e.g., carotenoids, whose presence was confirmed in *R. idaeus* [56,57]. The explanation for this fact lies in the different mechanisms of determining the antioxidant properties of compounds present in plant extracts using the ABTS and DPPH assays. More specifically, the ABTS test takes into account both hydrophobic and hydrophilic compounds, whereas the DPPH test takes into account only hydrophobic compounds. This latter mechanism also explains the low activity of the *R. occidentalis*/*R. idaeus* R1613412 hybrid fruits in the DPPH assay. The high antioxidant activity measured in the FRAP assay for all the studied fruits of different cultivars and hybrids of *R. occidentalis* and *R. idaeus* is associated with the fact that this assay allows for the determination of the antioxidant properties of hydrophilic compounds [58]. Initially, it was thought that the FRAP assay could be used to determine both hydrophilic and hydrophobic antioxidants [59].

Crossbreeding varieties of species from the *Rubus* genus can result in an increase in anthocyanin content, as exemplified by the R1613412 hybrid, while simultaneously reducing the pool of other polyphenolic compounds. The consequence of this is the observed decrease in antioxidant activity noted in some assays (ABTS and DPPH). This will be related to differences in the antioxidant activity mechanism, meaning that fruits with high anthocyanin content and low levels of other polyphenols will primarily inhibit free radical reactions. However, their free radical scavenging properties will be weaker. On the other hand, the high content of anthocyanins will contribute to a stronger anti-inflammatory effect by inhibiting, among others, cyclooxygenases, PGE2 and TNFα.

The antioxidant properties of raspberry fruits are an important part of their anti-inflammatory properties. In an in vitro model using PMA (phorbol-12-myristate-13-acetate) to stimulate the production of reactive oxygen species (ROS) in murine J774 macrophages, it has been shown that the antioxidant effect of raspberries is associated with the regulation of NADPH oxidase activity [14].

Chemometric analysis enabled the separation of *R. idaeus* and *R. occidentalis* cultivars and classified the hybrid *R. idaeus*/*R. occidentalis* R1314701 as belonging to the *R. occidentalis* species (Figure 3). At the same time, two hybrids, *Rubus occidentalis*/*Rubus idaeus* R1613411 and *R. idaeus*/*R. occidentalis* R1613409, can be classified as a purple raspberry, also taking into account the fact that their pedigree includes a black raspberry (Figure 3). Similarly, the cultivar ‘Heban’, studied as a cultivar of *Rubus occidentalis*, was classified as a purple raspberry cultivar because its pedigree includes both purple raspberry and red raspberry. The Polish National List of Fruit Plant Varieties (Figure 3) includes a cultivar of the purple variety ‘Heban’. Purple raspberry (*Rubus* × *neglected* Peck) is a hybrid derived from the crossing of two species, *R. idaeus* subsp. *strigosus* × *R. occidentalis*. Spearman’s correlations confirm the correlations between the total polyphenol content and antioxidant activity in the DPPH, ABTS and FRAP tests, as well as the anthocyanin content and antioxidant activity in the ABTS and FRAP tests.

## 5. Conclusions

Crossbreeding species/cultivars of the *Rubus* genus may result in an increased content of anthocyanins, but on the other hand, it may lead to a reduction in free radical scavenging activity in the ABTS and DPPH tests. It has been confirmed that raspberry fruits exhibit a high level of activity in inhibiting free radical chain reactions in the FRAP test while showing variable antioxidant activity in the ABTS and DPPH free radical scavenging tests. Additionally, it was also confirmed, that black raspberry fruits, regardless of the cultivar, have a stronger antioxidant effect than red raspberry fruits. The observed differences in antioxidant activity of the studied raspberry fruits may be related to the presence of carotenoid complexes that have not yet been identified in detail. This will be the subject of our further work. A detailed analysis of the chemical composition of raspberry fruits, together with an assessment of their antioxidant properties, enables the selection of fruits with the best health-promoting parameters for the processing industry, especially in the context of preventing lifestyle diseases. Chemometric analysis can be an effective tool in determining the species affiliation of obtained hybrids and cultivars.

## Figures and Tables

**Table 1:** Characteristics of the analysed fruits from different raspberry species and cultivars or hybrids (parent plants, colour of fruit, origin).

Cultivar or Hybrids/Colour of Fruit	Origin of the Cultivar	Pedigree * op—Open Pollination
*R. occidentalis* ‘Jewel’/black	Brzezna/Poland—from Niwa Berry Breeding Ltd.	‘Dundee’ × (‘Bristol’ × ‘Dundee’)
*R. occidentalis* ‘Niwot’/black	Brzezna/Poland—from Niwa Berry Breeding Ltd.	A complex cross between two breeding clones from a natural environment of origin in the USA
*R. occidentalis* ‘Mac Black’/black	Brzezna/Poland—from Niwa Berry Breeding Ltd.	-
*R. occidentalis*/*R. idaeus* R1613411/Purple	Brzezna/Poland—from Niwa Berry Breeding Ltd.	‘Jewell’ × R121304 (‘Litacz’ (‘Bristol’ × op) × purple raspberry × op)
*R. idaeus*/*R. occidentalis* R1613409/dark purple	Brzezna/Poland—from Niwa Berry Breeding Ltd.	‘Jewell’ × R121304 (purple raspberry × op)
*R. occidentalis*/*R. idaeus* R1613412/black	Brzezna/Poland—from Niwa Berry Breeding Ltd.	‘Jewell’ × R121304 (‘Litacz’ × purple raspberry) × op
*R. idaeus*/ *R. occidentalis* R1314701/dark purple	Brzezna/Poland—from Niwa Berry Breeding Ltd.	‘Litacz’ × ‘Sokolica’
*R. occidentalis* ‘Heban’ (R139501)/dark purple	Brzezna/Poland—from Niwa Berry Breeding Ltd.	(purple raspberry × ‘Polka’) × op
*R. occidentalis* ‘Bristol’ A/black	Nałęczów/Poland from “Czarna malina” Barbara Rusiecka-Górniak’	-
*R. occidentalis* ‘Bristol’ B/black	Łaziska/Poland from “BiGrim” Grzegorz Maryniowski	-
*R. idaeus* R1616002/red	Brzezna/Poland—from Niwa Berry Breeding Ltd.	R1634401 × ‘Polana’
*R. idaeus* ‘Husaria’/red	Brzezna/Poland—from Niwa Berry Breeding Ltd.	R120701 × ‘Sokolica’
*R. idaeus* ‘Delniwa’/red	Brzezna/Poland—from Niwa Berry Breeding Ltd.	‘Polka’ × R1211101

* Data provided by the breeder Niwa Berry Breeding Ltd.

**Table 2:** Determined antioxidant potential using DPPH, FRAP and ABTS assays and contents of polyphenolic and anthocyanin compounds of tested fruits from red and black raspberry varieties.

Species/Variety	DPPH (mmol TE/g DW)	ABTS (mmol TE/g DW)	FRAP (mmol TE/g DW)	Content % of Anthocyanin	Content % of Polyphenolic Compounds	Polyphenols Without Anthocyanins
*R. occidentalis* ‘Bristol’ A ^a^	2.14 ± 0.25 ^gjklm^	1.24 ± 0.21 ^klm^	3.87 ± 0.82 ^klm^	1.37 ± 0.06 ^bcdegjklm^	4.9 ± 0.18 ^c–m^	3.53 ± 0.19 ^c–m^
*R. occidentalis* ‘Bristol’ B ^b^	1.95 ± 0.22 ^gklm^	1.24 ± 0.44 ^klm^	4.09 ± 0.09 ^hklm^	2.17 ± 0.11 ^klm^	4.63 ± 0.23 ^c–m^	3.26 ± 0.13 ^c–m^
*R. occidentalis* ‘Jewel’ ^c^	1.82 ± 0.44 ^gkm^	1.70 ± 0.11 ^klm^	3.21 ± 0.25 ^klm^	2.56 ± 0.17 ^hklm^	3.8 ± 0.21 ^abfhiklm^	1.24 ± 0.09 ^ab^
*R. occidentalis* ‘MacBlack’ ^d^	1.63 ± 0.47	1.61 ± 0.24 ^klm^	3.56 ± 0.28 ^klm^	2.89 ± 0.09 ^hklm^	3.72 ± 0.3 ^abfklm^	0.83 ± 0.04 ^abf^
*R. occidentalis* ‘Niwot’ ^e^	1.59 ± 0.20	2.12 ± 0.24 ^klm^	2.73 ± 0.36 ^m^	2.36 ± 0.05 ^klm^	3.70 ± 0.15 ^abfklm^	1.34 ± 0.05 ^abgm^
*R. occidentalis* ‘Heban’ ^f^	1.53 ± 0.22	0.97 ± 0.68 ^k^	2.47 ± 0.07 ^m^	1.67 ± 0.03 ^gklm^	3.84 ± 0.11 ^abcdegklm^	2.17 ± 0.21 ^ab^
*R. occidentalis*/*R. idaeus* R1613412 ^g^	1.03 ± 0.14 ^abci^	1.10 ± 0.32 ^k^	3.79 ± 0.23 ^klm^	3.66 ± 0.21 ^afhiklm^	4.16 ± 0.07 ^fhijklm^	0.5 ± 0.11 ^abefhi^
*Rubus occidentalis*/*Rubus idaeus* R1613411 ^h^	1.60 ± 0.20	0.71 ± 0.33 ^k^	2.18 ± 0.24 ^bklm^	1.17 ± 0.04 ^cdgklm^	2.98 ± 0.14 ^abcgklm^	1.81 ± 0.1 ^adgjlm^
*R. idaeus*/*R. occidentalis* R1314701 ^i^	1.95 ± 0.21 ^gklm^	2.31 ± 0.66 ^klm^	3.52 ± 0.29	1.57 ± 0.06 ^gklm^	3.05 ± 0.23 ^abcgklm^	1.48 ± 0.11 ^abgm^
*R. idaeus*/*R. occidentalis* R1613409 ^j^	1.16 ± 0.05 ^a^	0.75 ± 0.36 ^k^	2.68 ± 0.33 ^m^	2.19 ± 0.04 ^klm^	3.16 ± 0.21 ^abgklm^	0.97 ± 0.1 ^abf^
*R. idaeus* ‘Delniwa’ ^k^	0.97 ± 0.08 ^abci^	0.27 ± 0.07 ^a–j^	1.05 ± 0.09 ^a–g^	0.6 ± 0.08 ^a–j^	1.76 ± 0.16 ^a–j^	1.16 ± 0.03 ^ab^
*R. idaeus* ‘Husaria’ ^l^	1.10 ± 0.22 ^abi^	0.54 ± 0.13 ^e^	1.49± 0.81	0.57 ± 0.06 ^a–j^	1.56 ± 0.2 ^a–j^	0.99 ± 0.07 ^abf^
*R. idaeus* ‘002′ ^m^	1.02 ± 0.09 ^abci^	0.55 ± 0.14 ^abcdei^	1.03± 0.04 ^a–j^	0.59 ± 0.03 ^a–j^	1.15 ± 0.09 ^a–j^	0.56 ± 0.08 ^abefhi^

Expressed as mean + SD (n = 3) (%) dry weight, superscript letters (a–m) within the same column identify groups of varieties showing statistically significant differences in content (*p* < 0.05) and statistically significant differences are observed between varieties marked with the same letter.

**Table 3:** Results of the Dunn’s test conducted for the analysed data matrix concerning raspberry species (*p* < 0.05, *p* < 0.01 and *p* < 0.001).

	*R. idaeus*	*R. occidentalis*/ *R. idaeus*	*R. occidentalis*	*R. idaeus*/ *R. occidenatlis*
***R. idaeus***	-	TPC (spectrophotometric) ^a^, FRAP ^a^, cyanidin 3-*O*-xylosyl rutinoside ^a^, anthocyanins (HPLC) ^b^, anthocyanins (HPTLC) ^b^, anthocyanins (spectrophotometric) ^b^, cyanidin 3-*O*-rutinoside ^b^, cyanidin 3-*O*-glucoside ^b^, pelargonidin 3-*O*-rutinoside ^c^, cyanidin 3-*O*-sambubioside ^c^	cyanidin 3-*O*-sambubioside ^b^, anthocyanins (HPLC) ^c^, anthocyanins (HPTLC) ^c^, anthocyanins (spectrophotometric) ^c^, TPC (spectrophotometric) ^c^, DPPH ^c^, ABTS ^c^, FRAP ^c^, cyanidin 3-*O*-rutinoside^c^, cyanidin 3-*O*-xylosyl rutinoside ^c^, cyanidin 3-*O*-glucoside ^c^, pelargonidin 3-*O*-rutinoside ^c^	
***R. occidentalis***/ ***R. idaeus***	TPC (spectrophotometric) ^a^, FRAP ^a^, cyanidin 3-*O*-xylosyl rutinoside ^a^, anthocyanins (HPLC) ^b^, anthocyanins (HPTLC) ^b^, anthocyanins (spectrophotometric) ^b^, cyanidin 3-*O*-rutinoside ^b^, cyanidin 3-*O*-glucoside ^b^, pelargonidin 3-*O*-rutinoside ^c^, cyanidin 3-*O*-sambubioside ^c^	-		
***R. occidentalis***	cyanidin 3-*O*-sambubioside ^b^, anthocyanins (HPLC) ^c^, anthocyanins (HPTLC) ^c^, anthocyanins (spectrophotometric) ^c^, TPC (spectrophotometric) ^c^, DPPH ^c^, ABTS ^c^, FRAP ^c^, cyanidin 3-*O*-rutinoside ^c^, cyanidin 3-*O*-xylosyl rutinoside ^c^, cyanidin 3-*O*-glucoside ^c^, pelargonidin 3-*O*-rutinoside ^c^		-	
***R. idaeus***/ ***R. occidenatlis***				-

^a^ *p* < 0.05, ^b^ *p* < 0.01, ^c^ *p* < 0.001.

## Data Availability

The datasets presented in this article are not readily available because the data are part of an ongoing study. Requests to access the datasets should be directed to miroslawa.krauze-baranowska@gumed.edu.pl.
